# Esophageal cancer practice guidelines 2022 edited by the Japan Esophageal Society: part 2

**DOI:** 10.1007/s10388-023-00994-1

**Published:** 2023-03-30

**Authors:** Yuko Kitagawa, Ryu Ishihara, Hitoshi Ishikawa, Yoshinori Ito, Takashi Oyama, Tsuneo Oyama, Ken Kato, Hiroyuki Kato, Hirofumi Kawakubo, Hiroshi Kawachi, Shiko Kuribayashi, Koji Kono, Takashi Kojima, Hiroya Takeuchi, Takahiro Tsushima, Yasushi Toh, Kenji Nemoto, Eisuke Booka, Tomoki Makino, Satoru Matsuda, Hisahiro Matsubara, Masayuki Mano, Keiko Minashi, Tatsuya Miyazaki, Manabu Muto, Taiki Yamaji, Tomoki Yamatsuji, Masahiro Yoshida

**Affiliations:** 1 Department of Surgery, Keio University School of Medicine, 35 Shinanomachi, Shinjuku-ku, Tokyo, 160-8582 Japan; 2 Department of Gastrointestinal Oncology, Osaka International Cancer Institute, Osaka, Japan; 3 QST Hospital, National Institutes for Quantum Science and Technology, Chiba, Japan; 4 Department of Radiation Oncology, Showa University School of Medicine, Tokyo, Japan; 5 Department of Hepato-Biliary-Pancreatic and Gastrointestinal Surgery, International University of Health and Welfare School of Medicine, Chiba, Japan; 6 Department of Endoscopy, Saku Central Hospital Advanced Care Center, Nagano, Japan; 7 Department Head and Neck, Esophageal Medical Oncology, National Cancer Center Hospital, Tokyo, Japan; 8 Kiryu Kosei General Hospital, Gunma, Japan; 9 Department of Pathology, Cancer Institute Hospital, Japanese Foundation for Cancer Research, Tokyo, Japan; 10 Department of Gastroenterology and Hepatology, Gunma University Graduate School of Medicine, Gunma, Japan; 11 Department of Gastrointestinal Tract Surgery, Fukushima Medical University, Fukushima, Japan; 12 Department of Gastroenterology and Gastrointestinal Oncology, National Cancer Center Hospital East, Chiba, Japan; 13 Department of Surgery, Hamamatsu University School of Medicine, Shizuoka, Japan; 14 Division of Gastrointestinal Oncology, Shizuoka Cancer Center, Shizuoka, Japan; 15 National Hospital Organization Kyushu Cancer Center, Fukuoka, Japan; 16 Department of Radiology, Yamagata University Graduate School of Medicine, Yamagata, Japan; 17 Department of Gastroenterological Surgery, Graduate School of Medicine, Osaka University, Osaka, Japan; 18 Department of Frontier Surgery, Graduate School of Medicine, Chiba University, Chiba, Japan; 19 Department of Central Laboratory and Surgical Pathology, National Hospital Organization Osaka National Hospital, Osaka, Japan; 20 Clinical Trial Promotion Department, Chiba Cancer Center, Chiba, Japan; 21 Department of Surgery, Japanese Red Cross Maebashi Hospital, Gunma, Japan; 22 Department of Clinical Oncology, Kyoto University Hospital, Kyoto, Japan; 23 Division of Epidemiology, National Cancer Center Institute for Cancer Control, Tokyo, Japan; 24 Department of General Surgery, Kawasaki Medical School, Okayama, Japan; 25 Department of Hepato-Biliary-Pancreatic and Gastrointestinal Surgery, School of Medicine, International University of Health and Welfare Ichikawa Hospital, Chiba, Japan

**Keywords:** Practice guidelines, Esophagus, Cancer

## Endoscopic surveillance

### Summary

In patients in whom complete response to definitive chemoradiotherapy for esophageal cancer has been achieved, computed tomography (CT) or endoscopic surveillance should be conducted to detect recurrence. Since intraesophageal recurrence, if detected early, is expected to be cured by salvage therapy, endoscopic surveillance is important from the standpoint of both the quality of life (QOL) and the prognosis.

Even after complete response of the primary lesion to definitive chemoradiotherapy has been achieved, recurrence of the primary lesion occurs at a high incidence. Therefore, it is important to perform endoscopy 1 month after complete response is documented and frequently (every 2–3 months) thereafter for 1 year, and subsequently, every 4–6 months, for early detection of any local recurrence of the primary lesion. Even if a complete response is not achieved, frequent endoscopy immediately after treatment is important, so that remnant lesions or relapse could be detected early and treated by endoscopic means or surgery.

## Surgical treatment

### Surgery for cervical esophageal carcinoma

#### Summary

In the treatment of cervical esophageal carcinoma, simultaneous laryngectomy is often required; therefore, preoperative chemoradiotherapy or definitive chemoradiotherapy is often selected in an attempt to conserve the larynx. Larynx-preserving surgery enables conservation of vocal functions, although it is associated with an increased risk of aspiration and pneumonia, necessitating the need for caution while selecting this treatment. Lowering of the QOL due to the loss of vocal functions poses a serious problem in patients who have undergone combined laryngectomy. No significant difference in the post-treatment prognosis has been reported so far between cervical esophageal carcinoma patients treated by surgery and by definitive chemoradiotherapy. The appropriate treatment in these patients should be selected with due consideration given to the QOL, etc.

#### General remarks

Since the trachea, large blood vessels, nerves, and the thyroid are present around the cervical esophagus, cervical esophageal carcinoma, which develops in this region, is frequently associated with malignant invasion of these adjacent organs. Lymph node metastasis is also frequently encountered; therefore, it is not uncommon for the malignancy to be at an advanced stage at diagnosis. There are a significant number of cases in which surgery is indicated, inasmuch as metastasis to the mediastinal lymph nodes, excluding some superior mediastinal lymph nodes ([105], [106rec]), is uncommon. A major problem with surgery for cervical esophageal cancer is that simultaneous laryngectomy is also indicated in many cases. Under these circumstances, chemoradiotherapy may be administered prior to surgery for tumor shrinkage, in an effort to preserve the larynx, or definitive chemoradiotherapy may be administered, followed by salvage surgery in the event of detection of remnant disease or recurrence.

Larynx-preserving surgery is indicated for patients in whom the tumor has not invaded the pharynx, larynx, or trachea. Conservation of the vocal functions is the utmost benefit of this treatment option, although it is associated with the risk of aspiration or pneumonia; not uncommonly, primary tracheotomy is required. Therefore, sufficient consideration should be given as to the indication for and choice of the operative procedure.

Combined laryngectomy (laryngopharyngoesophagectomy) is indicated for patients with tumors invading the pharynx, larynx, and trachea. The procedure may even be indicated for patients without direct pharyngeal invasion, in whom sufficient preservation of the esophagus to perform anastomosis with the organ graft is difficult. Marked lowering of the postoperative QOL due to loss of vocal functions poses a serious problem in patients who have undergone combined laryngectomy. Therefore, adoption of this treatment should be carefully determined considering the curability.

Reconstruction after surgical resection of cervical esophageal carcinoma is frequently performed using a free jejunal graft [1] or a gastric tube [2]. The method of first choice is reconstruction using a free jejunal graft, although reconstruction using a gastric tube is chosen for cases with thoracic esophageal cancer or cases in which the cervical esophageal cancer extends caudad to involve the thoracic esophagus, making caudal anastomosis difficult.

The frequency of lymph node metastasis in cases of cervical esophageal cancer is high, although it is confined in most cases to the cervical region and a part of the upper mediastinum ([105], [106rec]); therefore, lymph node dissection is primarily targeted at lymph nodes accessible through a cervical approach. Nevertheless, reports on the outcomes of lymphadenectomy in patients with cervical esophageal cancer are few as yet, and further investigation is needed.

No clear significant difference in the post-treatment prognosis has been reported until date between cervical esophageal carcinoma patients treated by surgery alone and those treated by definitive chemoradiotherapy. Selection among the available treatment options should be made with due consideration given to the post-treatment QOL, etc.

## Surgery for thoracic esophageal carcinoma

### Summary

Thoracic esophageal carcinoma is often associated with extensive lymph node metastasis in the cervical, thoracic, and abdominal regions, requiring three-field lymphadenectomy. In addition to conventional right thoracotomy/laparotomy, thoracoscopic surgery, laparoscopic surgery, robot-assisted surgery, mediastinoscopic surgery, and other novel procedures have recently been introduced, although further studies are required to establish their efficacy and safety.

### General remarks

Thoracic esophageal carcinoma is frequently associated with extensive lymph node metastasis in the cervical (cervical paraesophageal/supraclavicular), upper/middle/lower mediastinal (in particular, the bilateral para-recurrent laryngeal nerves), and upper abdominal (gastric lesser curvature) regions, and three-field lymphadenectomy covering the cervical, thoracic, and abdominal regions has been used in Japan. The 12th Edition of the Japanese Classification of Esophageal Cancer (2022) defines two-field lymphadenectomy as D2 and three-field lymphadenectomy as D3.

The basic surgical procedure for esophageal cancer consists of right thoracotomy/laparotomy, subtotal esophagectomy, lymph node dissection, gastric tube reconstruction, and cervical esophagogastric anastomosis. Left thoracotomy or transhiatal esophagectomy should also be considered depending on the tumor location, depth of invasion, and preoperative condition. The stomach is the first organ of choice for reconstruction, followed by the colon and small intestine. There are three reconstruction routes: ante-thoracic, retrosternal, and posterior mediastinal routes. Each route has its own advantages and disadvantages, and the appropriate route should be selected taking into account the patient characteristics and stage of the disease.

Recently, endoscopic surgery using a thoracoscope or laparoscope has been increasingly performed; however, there is little evidence to indicate the safety or long-term outcomes of such surgery. According to a randomized controlled study comparing thoracotomy/laparotomy and thoracotomy/laparoscopic surgery in France (MIRO trial), the incidence of postoperative respiratory complications was significantly lower in the laparoscopic surgery group than in the laparotomy group and the 3-year overall survival rate also tended to be higher in the laparoscopic surgery group (67% vs. 55%) [3].

As for thoracoscopic surgery, a randomized controlled study conducted in Europe, mainly in the Netherlands (TIME trial), reported that the incidence of postoperative pneumonia was significantly lower in cases treated by thoracoscopic surgery than in those treated by thoracotomy (12% vs. 34%), although no significant difference in the long-term outcomes between the two groups was observed [4, 5]. Currently, a Japanese randomized controlled study (JCOG1409) is ongoing to evaluate the safety and efficacy of this type of surgery, including the long-term outcomes. Robot-assisted esophagectomy began to be covered by the National Health Insurance in April 2018, although it is performed only in a limited number of institutions. Mediastinoscopic esophagectomy, which can be performed without thoracotomy, has also drawn attention. Robot-assisted surgery and mediastinoscopic surgery are expected to be minimally invasive, and further studies are required to objectively compare these procedures with thoracoscopic surgery.

Surgical complications include respiratory complications such as pneumonia, anastomotic leakage, and recurrent laryngeal nerve paralysis, and various improvements have been made to reduce the incidence of complications.

## Surgery for carcinoma of the esophagogastric junction

### Summary

There is no unanimity of opinion as to treatment policy and surgical procedures for carcinoma of the esophagogastric junction, particularly adenocarcinoma according to Nishi’s classification or Siewert type II carcinoma. The Japan Esophageal Society–Japanese Gastric Cancer Association Joint Working Group proposed the optimal extent of lymph node resection and surgical approach by the length of esophageal invasion, based on the metastasis rate to each lymph node group in patients who underwent resection for carcinoma of the esophagogastric junction in a prospective study.

### General remarks

As for the definition of carcinoma of the esophagogastric junction, Siewert’s classification is used overseas, whereas in Japan, Nishi’s classification is adopted by both the Japan Esophageal Society and the Japanese Gastric Cancer Association. In Siewert’s classification, type I lesions are often handled as carcinomas of the thoracic esophagus and type III lesions as cardiac carcinomas. Opinions are still divided as to the treatment policy and surgical procedures for adenocarcinomas according to Nishi’s classification and Siewert type II carcinoma.

**Fig. 1:**
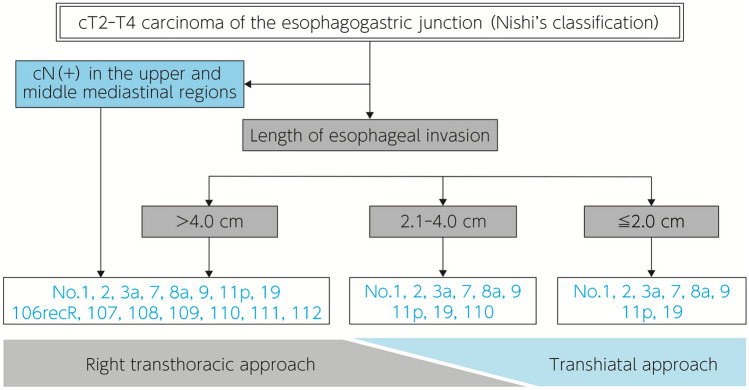
Algorithm for surgical approach to adenocarcinoma of the esophagogastric junction and lymph node dissection (modified from Kurokawa et al.: Ann Surg. 2021; 274(1): 120–127, Fig. 3)

Carcinoma of the esophagogastric junction may be associated with extensive lymph node metastasis involving the cervical region, mediastinum, upper abdomen, and areas circumjacent to the abdominal aorta, and no unified view has been reached in regard to the appropriate extent of lymph node dissection. The Japan Esophageal Society–Japanese Gastric Cancer Association Joint Working Group has laid down recommendations in respect of the extent of lymphadenectomy on the grounds of the dissection effect index (rate of metastasis × 5-year overall survival rate of patients with metastasis) derived from a retrospective analysis of the data of surgically treated cases. Nevertheless, the problems with retrospective analysis of tumors are that the patients are confined to those with tumors measuring ≤ 4 cm in diameter and that the subject population includes only a small number of cases with dissection of the lymph nodes in the upper and middle mediastinal regions and areas circumjacent to the abdominal aorta. A prospective study subsequently conducted by the Japan Esophageal Society–Japanese Gastric Cancer Association Joint Working Group proposed that [110] lymph node dissection and dissection of [106R] lymph node or middle/lower mediastinal lymph nodes through a (right) thoracic approach should be considered when the length of esophageal invasion is > 2 cm and > 4 cm, respectively, based on the metastasis rate to each lymph node group in patients who underwent resection for cT2-T4 carcinoma of the esophagogastric junction (Nishi’s classification) (Fig. 1).

The Japan Esophageal Society–Japanese Gastric Cancer Association Joint Working Group has proposed a definition of the esophagogastric junction based on endoscopic findings. In the clinical practice setting, however, the junction can scarcely be identified by endoscopy in cases of advanced carcinoma, and the frequent, concurrent hiatal herniation interferes with positional estimation of the junction even by an upper gastrointestinal series or CT. Thus, it may be said that only but an obscure judgment about the location of the junction can be obtained in the clinical setting. The extent of resection of the esophagus and stomach is determined in accordance with the resection margin of the main lesion and the extent of lymph node dissection, and the range of operative procedures available extend from total esophagogastrectomy to lower-third esophagectomy plus proximal gastrectomy. In surgery for carcinoma of the esophagogastric junction, the surgical invasiveness is affected not only by the extent of resection, but also by the surgical approach; therefore, the treatment selection must be approached by taking into consideration the balance between the surgical invasiveness and curability of the adopted procedure.

## Perioperative management and clinical path

### Summary

Appropriate perioperative management to prevent postoperative complications is important to promote safe team care. Although many institutions have introduced a clinical path for perioperative management of esophageal cancer patients, the method varies among institutions and its usefulness has not been established. In Europe and the United States, Enhanced Recovery after Surgery (ERAS)/fast-track surgery has been introduced as a new perioperative management program for many surgical procedures, and it has been shown to reduce the postoperative complications after esophagectomy and shorten the length of hospitalization. Patients with esophageal cancer often have malnutrition, and the ERAS guidelines state that nutritional assessment and enteral nutrition reduce the risk of postoperative complications. Postoperative rehabilitation aimed at early ambulation is provided at many institutions, and the possibility of preoperative rehabilitation intervention reducing the risk of postoperative complications was investigated.

### General remarks

Reduction in the invasiveness of esophageal cancer surgery from highly invasive thoracotomy/laparotomy has recently been attempted by introducing endoscopic surgery and robot-assisted surgery [6, 7]. In parallel with advances in surgical techniques, various perioperative management methods have been introduced to reduce the risk of postoperative complications. Appropriate perioperative interventions after accurate assessment of the surgical tolerability and sufficient risk management in elderly patients with various comorbidities/a history of smoking/drinking is very important to promote safe team medical care involving medical professionals from a variety of fields.

A clinical path is a standard medical practice plan containing information on the patient’s condition, goals of medical practice, and relevant evaluations and records, and represents a procedure for improving the quality of medical care through analysis of deviations from the standard. Although many institutions have introduced a clinical path for perioperative management of patients with esophageal cancer and its usefulness has been evaluated, the clinical path method varies among institutions in Japan and no large-scale prospective studies have been conducted. In Europe and the United States, the concept of ERAS or fast-track surgery has been introduced for perioperative management after many surgical procedures. The ERAS Group of the European Society for Clinical Nutrition and Metabolism (ESPEN) published an ERAS protocol for colectomy in 2004, which has since been applied for perioperative management in various surgeries [8]. Evaluation of the clinical efficacy of ERAS for esophagectomy showed that it reduces the incidence of respiratory complications and anastomotic leakage and shortens the length of hospitalization [9–11]. The previous edition of the Guidelines for Diagnosis and Treatment of Esophageal Cancer [12, 13] examined the significance of clinical paths in the perioperative management of esophageal cancer patients; however, there were few reports on classical clinical paths, and most reports had evaluated the effect of ERAS and fast-track surgery. Introduction of a clinical path in the perioperative management of esophageal cancer patients may reduce respiratory complications and other risks, but there is still limited evidence [12, 13]. From now on, the clinical significance of ERAS/fast-track surgery as a perioperative management program needs to be verified, and perioperative management procedures important for team medical care, such as rehabilitation and nutritional management, including the assessment of sarcopenia and frailty, need to be investigated for application to clinical practice.

Rehabilitation aimed at early ambulation has been provided to patients after surgery for esophageal cancer at many institutions. Postoperative rehabilitation is an important component of ERAS, which strongly recommends early ambulation to prevent postoperative complications [10]. Recently, sarcopenia in cancer patients has been found to not only be a postoperative complication, but also one of the prognostic factors, and the significance of preoperative rehabilitation intervention for this condition has been under debate. The Clinical Practice Guidelines for Cancer Rehabilitation of the Japanese Association of Rehabilitation Medicine only weakly recommends preoperative exercise therapy and respiratory rehabilitation, which are among the preoperative rehabilitation programs for gastrointestinal carcinoma, for patients with esophageal cancer [14]. The previous edition of the Guidelines for Diagnosis and Treatment of Esophageal Cancer also examined the usefulness of preoperative respiratory rehabilitation and showed that it may reduce the risk of postoperative respiratory complications [12, 13]. However, the specific intervention methods adopted and patient characteristics varied greatly, and the significance of rehabilitation as a perioperative management strategy was examined again for the latest edition of the guidelines.

Patients with advanced esophageal cancer often have malnutrition with tumor progression, and therefore are in strong need for perioperative nutritional management. The ESPEN [15] and ERAS [10] guidelines require preoperative nutritional assessment and early nutritional intervention and show that early enteral nutrition after surgery reduces the risk of postoperative complications. Immunonutrition containing omega-3 fatty acids or arginine has been reported to be useful, although the evidence is currently limited [10, 16]. Preoperative nutrition therapy combined with rehabilitation to improve sarcopenia has also been reported; however, the procedures adopted and facilities available vary widely, and no large-scale prospective studies have been conducted [17].

In recent years, the importance of perioperative oral care has been drawing attention in Japan. Although there are reports suggesting that perioperative oral care for esophageal cancer may prevent postoperative pneumonia [18], no large-scale comparative studies have been conducted, and this is an issue for future investigation.

## Chemotherapy/radiotherapy

### Preoperative/postoperative adjuvant therapy

#### Summary

Advanced esophageal cancer cannot be fully controlled by surgery alone, and the usefulness of adjuvant therapy has been explored. Preoperative cisplatin + 5-FU (CF) therapy had been the standard treatment, based on the results of the JCOG9907 study; however, the JCOG1109 study revealed a significant prolongation of the survival in the preoperative docetaxel + cisplatin + 5-FU (DCF) therapy group as compared with that in the preoperative CF group, and preoperative DCF therapy is now considered as the new standard treatment. Since preoperative treatment is associated with an increased frequency of toxicity, preventive measures against adverse events should be taken and appropriate selection of patients should be undertaken.

The Checkmate-577 study, which demonstrated the usefulness of postoperative nivolumab therapy, showed that 1-year administration of nivolumab prolonged the disease-free survival in patients who did not show pathologic complete response to surgery. Patients with adenocarcinoma or squamous cell carcinoma who received preoperative chemoradiotherapy were enrolled in this study, and the efficacy of this treatment is unknown in patients who have received preoperative chemotherapy, which is the standard treatment in Japan; therefore, postoperative nivolumab therapy should be administered only after carefully taking into account the risk–benefit balance.

### General remarks

In recent years, multidisciplinary treatment, including chemotherapy, radiotherapy, and surgery, has been used for esophageal cancer. The JCOG9204 study conducted in Japan (1992–1997) compared the outcomes of surgery alone with the outcomes of surgery plus postoperative chemotherapy with CF [19]. While no significant difference in the overall survival was observed between the two groups, the 5-year disease-free survival (DFS) was significantly better in the surgery plus postoperative chemotherapy group (55%) than in the surgery-alone group (45%); furthermore, this improved prognosis was particularly evident in the pathological lymph node metastasis-positive cases. As a result, surgery plus postoperative chemotherapy became the standard treatment in Japan for patients with lymph node metastasis diagnosed by histopathology after surgical resection. Subsequently, the JCOG9907 study (1999–2006) investigated the optimal timing, in relation to surgery, of adjuvant chemotherapy with CF, and showed that the 5-year overall survival was significantly better in the preoperative chemotherapy plus surgery group (55%) than in the surgery plus postoperative chemotherapy group (43%) [20]. Thereafter, preoperative adjuvant chemotherapy with CF followed by radical surgery came to be adopted as the standard of care for patients with resectable cStage II and III thoracic esophageal cancer in Japan.

On the other hand, in Europe and North America, preoperative chemoradiotherapy followed by radical surgery is actively used. Preoperative chemoradiotherapy yields a higher local control rate (pathologic complete response [pCR] rate) than preoperative chemotherapy alone, but is also thought to increase the risk of perioperative complications and surgery-related mortality. In Japan, local control by accurate lymph node dissection during surgery has been pursued, and preoperative radiotherapy has been thought to be harmful and not necessarily beneficial. In Europe and North America, several randomized controlled studies investigating the usefulness of preoperative chemoradiotherapy have been reported [21], because surgery is thought to have limitations in local control. The CROSS trial, which is a large-scale randomized controlled study conducted in the Netherlands, showed that the overall survival was significantly longer in the preoperative chemoradiotherapy + surgery group than in the surgery alone group (median overall survival, 49.4 vs. 24.0 months) [22]. On the other hand, there were no significant differences in the incidence of postoperative complications between the two groups.

Recently, immune checkpoint inhibitors that have been reported to be effective against advanced/recurrent esophageal cancer were evaluated as adjuvant therapy after surgery for esophageal cancer. In patients with esophageal squamous cell carcinoma or adenocarcinoma who underwent R0 resection after preoperative chemoradiotherapy, but failed to show pCR, the DFS, which was the primary endpoint of the study, was significantly longer in the postoperative 1-year nivolumab treatment group than that in the placebo group (median DFS, 23.0 vs. 11.0 months; hazard ratio [HR]: 0.69 [95% confidence interval (CI) 0.56–0.86]). In this study, 70% of the subjects had adenocarcinoma and many subjects were from overseas; however, a significant difference in the disease-free survival was also observed in a subgroup of patients with squamous cell carcinoma [23].

A subgroup analysis in the JCOG9907 study revealed that preoperative chemotherapy may be poorly effective in patients with Stage III disease, and the development of more effective treatment modalities has been desired. Phase II studies have demonstrated the short-term efficacy of DCF therapy, in which docetaxel is added to CF therapy, and of preoperative chemoradiotherapy, which is used overseas, and the long-term efficacy of these modalities was investigated.

The JCOG1109 study is a randomized controlled study performed to confirm the superiority of preoperative DCF therapy and preoperative chemoradiotherapy (CF, radiotherapy at 41.4 Gy) over the currently used preoperative CF therapy, and the results of the study were reported in January 2022. Preoperative DCF therapy provided more prolonged survival than the conventional standard preoperative CF therapy (3-year overall survival rate: 62.6% in the preoperative CF therapy group vs. 72.1% in the preoperative DCF therapy group) (HR 0.68 [95% CI 0.50–0.92]). Patients who received preoperative chemoradiotherapy showed a 3-year survival rate of 68.3%, but did not show significantly longer survival than those who received preoperative CF therapy (HR 0.84 [95% CI 0.63–1.12]). As for perioperative complications, the incidence of ≥ Grade 2 adverse events was significantly lower in the preoperative DCF group [24]. Based on these results, preoperative DCF therapy came to be considered as the standard treatment for patients with locally advanced, resectable esophageal squamous cell carcinoma.

## Chemoradiotherapy

### Summary

Chemoradiotherapy has been demonstrated to provide more prolonged survival than radiotherapy alone in patients with locally advanced esophageal cancer. It is considered as the standard of care in non-surgical treatment, and chemoradiotherapy aimed at complete cure is indicated for cStage 0 to IVA cancer. A parallel group comparative study (JCOG0502 Study) showed that outcomes of chemoradiotherapy were not inferior to those of surgery in patients with cStage I disease. No studies have directly compared chemoradiotherapy and surgery in patients with cStage II or III cancer, and chemoradiotherapy is considered as one of the treatment options in patients who do not wish to undergo surgery as the initial treatment. It is important to select the appropriate radiation dose, irradiation area, and chemotherapy regimen while considering the most suitable treatment strategy, and also to consider the salvage treatments for remnant and recurrent lesions after chemoradiotherapy.

### General remarks

#### Chemoradiotherapy for cStage 0 and I disease

Chemoradiotherapy is indicated for lesions covering ≥ 3/4th of the circumference, which are difficult to treat endoscopically, and those invading up to the submucosa or deeper. The JCOG0502 study showed good results of chemoradiotherapy, with a complete response rate of 87.3% and 5-year overall survival rate of 85.5%, which were not inferior to those of surgery, although it was not a randomized controlled study [25]. Twenty patients (12.7%) had remnant cancer and 48 (30.2%) developed recurrence after treatment, but many of these lesions could be completely cured by endoscopic treatment or surgical resection. cStage I patients are known to develop recurrent or metachronous multiple lesions in the esophagus after showing complete response [26], and it is important to perform CT and endoscopy every 3–4 months for at least 2 years after complete response is obtained, and every 6 months thereafter, for detecting recurrent or metachronous multiple lesions at a sufficiently early stage so that the lesions can be treated endoscopically.

In addition, it has been reported that 10–50% of patients with obvious submucosal invasion or intramucosal lesions with vascular invasion after endoscopic treatment develop lymph node metastasis, and these patients were likely to have non-curative resection [27]. For additional treatment of these patients, radical surgery with lymph node dissection is currently used as the standard of care, while one report has suggested the usefulness of prophylactic chemoradiotherapy in combination with CF for regional lymph node metastasis [28]. In the JCOG0508 study, cT1bN0 esophageal cancer with a limited depth of invasion (up to SM2), which was estimated to be treatable endoscopically, was treated endoscopically, and patients with pathologically confirmed complete resection who had pT1a with positive vascular invasion or pT1b received prophylactic chemoradiotherapy. With such treatment, these patients showed a 3-year overall survival rate of 90.7% (90% CI 84.0–94.7) [29]. On the other hand, 3 (20%) of the 15 patients who had positive surgical margins after endoscopic treatment and received definitive chemoradiotherapy died of the disease. It should be carefully investigated as to which subpopulation of patients with cT1bN0 disease would be suitable candidates for this treatment.

#### Chemoradiotherapy for cStage II and III disease

The RTOG9405/INT0123 study conducted by the US RTOG compared cisplatin (75 mg/m^2^ on days 1 and 29) + 5-FU (1000 mg/m^2^ on days 1–4 and 29–32) chemotherapy with radiotherapy at a radiation dose of 50.4 Gy, and the same chemotherapy with radiotherapy at a radiation dose of 64.8 Gy, and revealed that while the survival was not prolonged any further, higher toxicity was obtained in the 64.8 Gy group [30]. Based on this, chemotherapy with cisplatin (75 mg/m^2^ on days 1 and 29) + 5-FU (1000 mg/m^2^ on days 1–4 and 29–32) combined with radiotherapy at a radiation dose of 50.4 Gy (RTOG regimen) is considered as one of the standard chemoradiotherapy treatment regimens.

The JCOG0909 study aimed at assessing the usefulness of definitive chemoradiotherapy followed by surgical intervention as salvage treatment if needed in patients with cStage II or III esophageal cancer showed good outcomes, with a complete response rate of 59%, 3-year overall survival rate of 74.2% [90% CI 65.9–80.8], 5-year overall survival rate of 64.5% [95% CI 53.9–73.3], 5-years recurrence-free survival rate of 48.3% [95% CI 37.9–58.0], 5-year esophagus preservation rate of 54.9% [95% CI 44.3–64.4] [31]. In the JCOG1109 study that assessed the efficacy of preoperative chemotherapy + surgery in patients with the same disease, patients treated with DCF therapy + surgery showed a 3-year survival rate of 72.1% [24]. The characteristics of the enrolled patients were different between the JCOG0909 and JCOG1109 studies (clinical stage: IIA/IIB/III [according to the UICC 6th edition] = 22/38/34 in the JCOG0909 study, IB/II/III [according to the UICC 7th edition] = 51/174/376 in the JCOG1109 study), and a direct comparison of the results would be unreasonable.

Definitive chemoradiotherapy is recommended as one of the treatment options for patients with cStage II or III esophageal cancer who do not wish to undergo surgery as initial treatment, and as a treatment with which complete cure can be expected for patients who are intolerant to surgery. However, adequate follow-up and active salvage therapy, which are described below, are important, and it is necessary to consider treatment strategies, including salvage treatments, after chemoradiotherapy.

#### Chemoradiotherapy for cStage IVA esophageal cancer

When a lesion that is not amenable to surgical resection is limited to the irradiation area, chemoradiotherapy is used as the standard treatment. A single-center phase II study of CF in combination with radiotherapy at a radiation dose of 60 Gy reported a complete response rate of 33% and 3-year overall survival rate of 23%, and a multicenter study, the JCOG9516 study, reported a complete response rate of 15% and 2-year overall survival rate of 31.5% [32, 33]. As a result, chemoradiotherapy with CF has come to be used as the standard treatment. Two randomized controlled studies comparing standard chemotherapy with 5-FU (700 mg/m^2^ on days 1–4 and 29–32) + cisplatin (70 mg/m^2^ on days 1 and 29) and low-dose chemotherapy with 5-FU (200 mg/m^2^) + cisplatin (4 mg/m^2^) on days 1–5, 8–12, 15–19, 22–26, 29–33, and 36–40, both combined with radiation at the dose of 60 Gy, failed to show any clear advantage of low-dose chemotherapy [34, 35].

A clinical study of chemoradiotherapy with DCF, in which docetaxel is added to CF, in combination with radiotherapy reported good results with a complete response rate of 42.1%; however, Grade 3–4 esophagitis/febrile neutropenia occurred in ≥ 30% of the subjects. Therefore, adoption of this treatment needs to be carefully considered [36]. Multidisciplinary treatment in which surgery or chemoradiotherapy is performed after intensive induction chemotherapy has been shown to yield good therapeutic results with a 3-year overall survival rate of 46.6% [37], and a comparative study (JCOG1510 study) comparing chemoradiotherapy with induction DCF therapy followed by conversion surgery is ongoing [15, 38].

#### Radiation dose and chemotherapy regimens used in chemoradiotherapy (Table 1)

**Table 1:** Summary of prospective clinical studies of chemoradiotherapy

Study name	cStage Histological type	Regimen	Radiation dose (Gy)	Complete response rate (%)	Survival (%)
JCOG0502 [25]	cStage Ib SCC	5-FU 700 mg/m^2^ day 1–4, 29–32 Cisplatin 70 mg/m^2^ day1, 29	60	87.3	5-year survival 85.5
RTOG85-01 [39]	cStage I, II, III SCC, AC	Radiotherapy alone	64	NA	5-year survival 0
		5-FU 1000 mg/m^2^ day 1–4, 29–32 Cisplatin 75 mg/m^2^ day1, 29	50	NA	5-year survival 26
RTOG94-05 [30]	cStage I, II, III SCC, AC	5-FU1000mg/m^2^ day 1–4, 29–32 Cisplatin 75 mg/m^2^ day 1, 29	50.4	NA	2-year survival 31
		5-FU1000mg/m^2^ day 1–4, 29–32 Cisplatin 75 mg/m^2^ day 1, 29	64.8	NA	2-year survival 40
JCOG0909 [31]	cStage II, III SCC	5-FU 1000 mg/m^2^ day 1–4, 29–32 Cisplatin 75 mg/m^2^ day 1, 29	50.4	59	3-year survival 74.2
JCOG9516 [33]	Unresectable local SCC	5-FU 700 mg/m^2^ day 1–4, 29–32 Cisplatin 70 mg/m^2^ day1, 29	60	15	2-year survival 31.5
JCOG0303 [35]	Unresectable local SCC	5-FU 700 mg/m^2^ day 1–4, 29–32 Cisplatin 70 mg/m^2^ day1, 29	60	0	1-year survival 55.9
		5-FU 200 mg/m^2^/5 doses weekly for 6 weeks Cisplatin 4 mg/m^2^/5 doses weekly for 6 weeks	60	1.4	1-year survival 56.3
KROSG0101/JROSG021 [34]	cStage II, IVA local SCC	5-FU 700 mg/m^2^ day 1–14, 29–42 Cisplatin 70 mg/m^2^ day 1–5, 8–12, 29–33, 36–40	60	NA	2-year survival 46
		5-FU 250 mg/m^2^ first 5 days of each week Cisplatin 4 mg/m^2^ before irradiation	60	NA	2-year survival 44
KDOG0501 [36]	Unresectable local SCC	5-FU 400 mg/m^2^ day 1–5, 15–19, 29–33 Cisplatin 40 mg/m^2^ day 1, 15, 29 Docetaxel 20-40 mg/m^2^ day 1, 15, 29	61.2	42.1	1-year survival 63.2

*SCC* squamous cell carcinoma, *AC* adenocarcinoma, *5-FU* 5-fluorouracil, *NA* not available

The RTOG8501 study recommended chemoradiotherapy as the standard treatment, because comparison of radiotherapy (64 Gy) alone and concurrent chemoradiotherapy (CF + 50 Gy) for esophageal cancer revealed significantly superior treatment outcomes of chemoradiotherapy [39].

In addition, a meta-analysis of studies of chemotherapy and radiotherapy reported that concurrent chemotherapy and radiotherapy provided significantly more prolonged survival than sequential chemotherapy and radiotherapy [40]. The above-mentioned RTOG9405/INT0123 study revealed no superior outcomes in terms of the survival or local control rate in the high-dose group, concluding that a radiation dose of 50.4 Gy should be used in combination with cisplatin (75 mg/m^2^ on days 1 and 29) + 5-FU (1000 mg/m^2^ on days 1–4 and 29–32) chemotherapy. The ARTDECO study, as well as the RTOG9405/INT0123 study, also revealed no superior outcomes in the high-dose group and concluded that a radiation dose of 50.4 Gy should be used [41].

In Japan, a regimen of cisplatin (70 mg/m^2^ on days 1 and 29) + 5-FU (700 mg/m^2^ on days 1–4 and 29–32) chemotherapy combined with a radiation dose of 60 Gy, which was adopted in the JCOG0502 and JCOG0303 studies, is widely used in patients with cStage I or IVA disease, while a regimen of cisplatin (75 mg/m^2^ on days 1 and 29) + 5-FU (1000 mg/m^2^ on days 1–4 and 29–32) chemotherapy combined with a radiation dose of 50.4 Gy, which was adopted in the JCOG0909 study, is generally used in patients with cStage II or III disease.

#### Adverse effects of definitive chemoradiotherapy

Adverse effects of chemoradiotherapy are mainly classified into acute and late toxicities. Acute toxicities occur mainly during concurrent chemotherapy and radiotherapy, within 1 to 2 months after the start of treatment. Late toxicities are often associated with radiation and occur a few months to a few years after completion of treatment. Some of the acute toxicities are gastrointestinal toxicity, including nausea and vomiting, renal impairment, leukopenia, esophagitis, and dysphagia, and should be treated according to guidelines such as the “Guidelines for Proper Use of Antiemetics” and “Practical Guideline of Febrile Neutropenia”. Some of the late toxicities are radiation pneumonitis, pleural effusion, pericardial effusion, constrictive pericarditis, hypothyroidism, and thoracic vertebral fracture, which interfere with daily life in approximately 10% of patients [42–44]. Since late toxicities may be fatal, regular follow-up, medical interviews to obtain information on subjective symptoms such as dyspnea, and early treatment are important. Recently, high-precision radiotherapy, such as intensity-modulated radiotherapy with X-rays and particle therapy with proton or heavy ion beams, has been reported to reduce these adverse effects [45–47].

#### Salvage treatment for local remnant/recurrent lesions after definitive chemoradiotherapy

When there is a local remnant or recurrent lesion after chemoradiotherapy for esophageal cancer, salvage surgery or endoscopic treatment may yield long-term survival. It has been reported that in salvage surgery, R0 resection allows long-term survival, but at the same time increases the incidence of postoperative complications and in-hospital mortality [48–52]. In the above-mentioned JCOG0909 study, 5% (5 of 94) and 29% (27 of 94) of the patients underwent endoscopic treatment and salvage esophagectomy, respectively, as salvage treatment. Of the 27 patients who underwent salvage esophagectomy, 5 (19%) had Grade 3–4 surgery-related complications and there was 1 surgery-related death; however, R0 surgery was achieved in 23 patients (85%). When a remnant lesion remains confined to the mucosa, salvage endoscopic treatment can be performed safely [53, 54]. Photodynamic therapy (PDT) has been reported to yield good results even in cases with suspected invasion of the submucosa or muscularis propria, and PDT is considered as one of the potentially useful treatment options [55].

## Radiotherapy alone

### Summary

For definitive radiotherapy, concurrent chemoradiotherapy is recommended; however, radiotherapy alone is often used when the use of chemotherapy is precluded by factors such as the presence of complications, advanced age, poor general condition, or other reasons. In addition, it is considered that unnecessary prolongation of the irradiation period should be avoided.

### General remarks

Randomized controlled studies and their meta-analyses have demonstrated that concurrent chemoradiotherapy is more effective than radiotherapy alone for definitive treatment of esophageal cancer [56, 57]. Therefore, definitive radiotherapy alone is indicated for patients in whom the use of chemotherapy is precluded by factors such as the presence of complications, advanced age, poor general condition, patient refusal, or other reasons.

As for the treatment outcomes of radiotherapy alone, according to an analysis of enrolled patients by the Japan Esophageal Society, the 5-year overall survival rates were 41.8%, 18.5%, 9.3%, and 13.9% in patients with cStage 0-I, II, III, and IV cancer, respectively [58]. A randomized phase II clinical study conducted in the era when a two-dimensional treatment plan was used showed that elderly patients aged ≥ 80 years treated by radiotherapy alone at 66 Gy/33 Fr showed a median survival of 30 months and a 3-year overall survival rate of 39% [59]. Even though use of chemotherapy may have been difficult in most of the patients, radiotherapy alone allowed long-term survival and cure in a certain proportion of patients.

When radiotherapy alone is undertaken, since the local control rate may decrease due to accelerated repopulation of the tumor cells during the irradiation period, it is considered that unnecessary prolongation of the total treatment duration should be avoided [60]. A total dose of 60–70 Gy, which is somewhat higher than the dose used for definitive chemoradiotherapy, is commonly prescribed.

## Follow-up after radical resection of esophageal cancer/treatment of recurrence

### Summary

The purpose of follow-up after radical resection of esophageal cancer is (1) to detect and treat recurrence early, (2) to provide systemic management and assess/improve the QOL in the short-to-medium/long term after completion of treatment, and (3) to detect and treat multiple/double cancers early. However, there are no reports providing high-level evidence of the follow-up method from the perspective of the survival rate, QOL improvement, examination costs, or adverse events. In Japan, there are very few institutions in which QOL is assessed in the medium-to-long term after radical resection of esophageal cancer. It is also important to pay attention to the possibility of occurrence of metachronous multiple esophageal cancers and double cancers in other organs. Establishment of a consensus-based follow-up system and verification of its effectiveness are required.

Patients with recurrence after radical resection for esophageal cancer have poor survival rates. Treatment varies depending on the pattern of recurrence (lymph node/local recurrence, distant organ recurrence, pleural and peritoneal recurrence, or mixed recurrence), whether recurrence is in the surgical area, and the general condition of the patient at the time of recurrence. In many reported cases, early detection and treatment have allowed long-term survival, depending on the number and extent of recurrent lesions; however, there is currently little high-level evidence of the efficacy of surgical treatment or (chemo-) radiotherapy aimed at complete cure for recurrent esophageal cancer. Treatment for suppressing the exacerbation of recurrent lesions or improving QOL is also often used, but there are few studies comparing the efficacy of such treatment with best supportive care.

### General remarks

#### Follow-up after radical resection

Many reports have shown that recurrence after radical resection of esophageal cancer occurs in about 40–60% of cases [61–65]. Although in approximately 80–90% of cases, the recurrences occur early, often within 2 years after surgery [62, 63, 66]; in some cases, they occur much later, and this possibility should be borne in mind. However, the actual method of follow-up after radical resection of esophageal cancer is currently determined by each institution [67, 68], and no studies have clarified the usefulness of regular follow-up or an effective method of follow-up.

The results of a nationwide survey of follow-up after radical treatment of esophageal cancer at institutions accredited by the Japan Esophageal Society, which was conducted by the Committee on Guidelines for Diagnosis and Treatment of Esophageal Cancer of the Japan Esophageal Society in 2020, are summarized below (only the results for patients with pStage II, III, or IV are shown; for details, see reference [68]). Among follow-up examinations, < medical interviews and physical findings > were performed ≥ 5 times a year in 64% of institutions in postoperative year 1, ≥ 3 times a year in 61% in year 5, and ≥ once a year in 76% in year 10; < cervical-pelvic CT > was performed ≥ 3 times a year in 61% in year 1, ≥ once a year in 96% in year 5, and ≥ once a year in 59% in year 10; < upper gastrointestinal endoscopy > was performed ≥ once a year in almost 100% up to year 5 and ≥ once a year in 74% even in year 10. On the other hand, QOL after radical resection was regularly assessed only in 13% of institutions even in postoperative year 1 and only in 3% in postoperative year 5. It seems necessary to raise awareness about the importance of postoperative QOL assessment in Japan [69].

In esophageal cancer cases, development of metachronous multiple cancers in the esophagus or metachronous cancer in other organs, such as gastric cancer and head and neck cancer, is not rare [70–72]. Bearing the possibility of development of multiple cancers and double cancers in mind, upper gastrointestinal endoscopy needs to be regularly performed to carefully observe the pharynx, remnant esophagus, and stomach (gastric tube). Furthermore, attention also needs to be paid to the possible development of colorectal and other cancers. In the above-mentioned nationwide survey [68], only approximately 30% of institutions regularly screened the patients for metachronous head and neck cancer even up to postoperative year 5.

#### Treatment of recurrence after radical resection

Recurrence after radical resection of esophageal cancer includes lymph node/local recurrence, distant organ recurrence, and pleural and peritoneal recurrence, and a mixture of these types of recurrence is also common. Although the incidence of these types of recurrence varies greatly among studies, recurrence in the neck/superior mediastinum is common in cases of lymph node recurrence, while in cases of distant organ recurrence, the lung, liver, bone, and brain are the more common sites of recurrence. Even metastasis to the small intestine and colon have been reported.

Treatment of recurrence after radical resection for esophageal cancer is selected according to the site, pattern, and extent of recurrence. Treatment would vary depending on the general condition of the patient at the time of recurrence, on whether the recurrence is in the surgical area, and on whether irradiation was given preoperatively or postoperatively. Therefore, there have been few reports of large-scale studies of the treatment outcomes according to various pathological conditions. Although patients with recurrence after radical resection for esophageal cancer have extremely poor survival rates, active treatment, such as resection of recurrent lesions or chemoradiotherapy, has allowed long-term survival or complete cure in many reported cases. Many studies have reported that particularly patients with a few metastatic lesions and those with cervical lymph node recurrence have a good prognosis [61–64, 73–79].

## Palliative treatment

### Summary

Palliative care should be commonly provided for all types of cancers, and all medical professionals involved in cancer care are required to have mastery over the basic knowledge and skills needed for providing palliative care. As for the psychological and mental aspects, some cancer patients develop psychiatric symptoms such as anxiety and depression, and it is sometimes necessary to refer such patients to mental health care specialists. In esophageal cancer patients, in particular, dysphagia, malnutrition, cough due to aspiration or fistula formation with the airways, and other symptoms often decrease the QOL, and provision of specific treatment to provide relief from these symptoms and maintaining/improving the QOL of the patients should be considered from even the early stages of treatment. However, the method of palliation adopted is determined by the prevailing practice at individual institutions, and further evaluation is required.

### General remarks

The WHO (2002) defines palliative care as “an approach that improves the QOL of patients and their families facing problems associated with life-threatening illness, through the prevention and relief of suffering by means of early identification and impeccable assessment and treatment of pain and other problems, physical, psychosocial and spiritual”. The Third Basic Plan to Promote Cancer Control Programs in fiscal year 2018 states that “promotion of palliative care from the time of cancer diagnosis” is an issue that needs attention. The above-mentioned palliative care is common to all cancer patients and is provided in daily practice; not only the attending physicians and nurses, but also palliative care specialists, psycho-oncologists, dentists, pharmacists, psychologists, certified social workers, care workers, rehabilitation technicians, and other professionals need to provide palliative care as a team. Many guidelines and manuals developed by the Japanese Society for Palliative Medicine, the Japanese Association of Supportive Care in Cancer, and other organizations provide useful information on palliative treatment and supportive care in cancer.

Patients with esophageal cancer often suffer from dysphagia and malnutrition due to esophageal obstruction, cough due to aspiration/fistula, and chest pain due to the tumor, resulting in a lowered QOL already at the time of diagnosis and require specific palliative treatment. Even while providing treatment for cure, it is important, from the early stage, to provide treatment for the relief of symptoms and for maintaining/improving the QOL of the patients [80]. As for the psychological and mental aspects, a survey using the Hospital Anxiety and Depression Scale, which is a tool for psychological assessment, in preoperative patients with esophageal cancer revealed that anxiety and depression scores were significantly higher in 34% and 23% of the patients, respectively [81], indicating that it is important to initiate provision of psychological support and mental health care preoperatively. Patients who cannot consume food for a long time due to esophageal cancer may develop psychiatric symptoms such as anxiety and depression, and it is important to provide an in-depth explanation of the clinical course and emotional support to these patients. In some cases, it is necessary to refer the patients to mental health care specialists.

In palliative treatment for patients with terminal esophageal cancer, problems that need to be handled, in particular, are dysphagia due to esophageal obstruction and malnutrition caused by dysphagia, symptoms caused by airway obstruction or fistula formation with the airways, cachexia and other symptoms due to distant metastases and hypercalcemia. To improve the symptoms of esophageal obstruction and airway obstruction and those caused by fistula, palliative radiotherapy, chemoradiotherapy, esophageal stenting, airway stenting, esophageal bypass surgery, and/or other treatments may be used [82–84]. In addition, aortic stenting may also be used to prevent or treat bleeding due to aorto-esophageal fistula [85].

Medical professionals involved in the treatment of esophageal cancer often encounter potentially fatal complications, such as sudden respiratory arrest due to airway obstruction and massive hematemesis due to aortic perforation. Once such complications occur, it is difficult to save the lives of the patients in most cases; therefore, it is important to provide a thorough explanation in advance, particularly to the patients’ families. Patients and their families are often forced to live in fear of sudden change/death, and psychological support and mental care for them are indispensable.

However, regarding the palliative treatment characteristic of esophageal cancer as described above, there are many fields where evidence is scarce and conducting clinical trials is not easy, so a clear policy has not been shown. In the future, it is hoped that the accumulation of experience and knowledge regarding alleviation of physical and psychological pain in esophageal cancer patients will lead to the enhancement of clinical question from a broader perspective and the development of clinical research that serves as the basis for it.

## Endoscopic stenting

### Summary

Patients with incurable esophageal cancer may develop various symptoms due to esophageal obstruction or fistula formation, resulting in a deteriorated QOL. Palliative (chemo-) radiotherapy, esophageal stenting, and other treatment modalities are used to improve the symptoms caused by esophageal obstruction or fistula.

A study comparing the effects of palliative radiotherapy and esophageal stenting for dysphagia caused by esophageal obstruction reported that palliative radiotherapy was associated with a lower incidence of adverse events and was more effective at providing relief from pain than stenting, while stenting improved dysphagia more rapidly [86]. When rapid improvement in dysphagia is desirable from the perspective of patient preferences or patient condition, esophageal stenting would be the best treatment option.

In incurable esophageal cancer patients presenting with cancerous obstruction after (chemo-) radiotherapy, esophageal stenting is one of the treatment options. Although stenting after radiotherapy is thought to increase the risk of adverse events, such as bleeding, fistula formation, and perforation, the use of a stent with a low radial force has been reported to allow relatively safe stenting. These points should be kept in mind when providing treatment. One of the options other than esophageal stenting is creation of a nutritional fistula to allow the patient to be switched to home care. Percutaneous endoscopic gastrostomy is very safe, and it may provide superior survival to stenting [86]. In cases in which percutaneous endoscopic gastrostomy is difficult due to severe obstruction that is difficult to negotiate even with a small-diameter endoscope, or in patients with a history of abdominal surgery, open gastrostomy or jejunostomy may be performed.

## Aortic stent grafting

### Summary

In locally advanced esophageal cancer patients with fistula formation with the aorta, aortic stent grafting could be a life-saving option. However, most of the reports on aortic stent grafting are case reports of a small number of patients, and the efficacy of aortic stent grafting has not yet been established. Furthermore, so far only case reports, each involving only a few patients, have shown the usefulness of prophylactic aortic stent grafting aimed at radical surgery for esophageal cancer invading the aorta, and further studies are expected to evaluate its efficacy.

### General remarks

Since esophageal carcinoma is anatomically adjacent to the thoracic aorta, progression of the disease could lead to aorto-esophageal fistula formation. The usefulness of aortic stent grafting as a treatment option for aorto-esophageal fistula has been evaluated. A search of the literature using the keywords “esophageal cancer”, “stent-graft”, “endovascular treatment”, and “aorto-esophageal fistula” yielded 22 PubMed articles and 12 ICHUSHI articles. All were reports of studies of a small number of patients, excluding 1 report of a retrospective study of accumulated cases and 1 report of a questionnaire survey conducted by the Japan Esophageal Society, which is described below [87, 88]. Aortic stent grafting was used for emergency hemostasis of bleeding from the aorto-esophageal fistula in many cases, but the clinical course varied (e.g., in one case, fistula formation was suspected by CT, and aortic stent grafting was used to prevent bleeding), making a quantitative systematic review difficult. As a result, there were a total of about 100 cases, and the purpose, i.e., hemostasis/bleeding prevention, was achieved in these cases, suggesting the efficacy of stent grafting.

In recent years, preoperative prophylactic stent grafting for locally advanced esophageal cancer invading the aorta has been reported. In four case reports retrieved by the literature search, it was shown to be effective in a total of 11 cases [89–91]. The results of a survey of thoracic stent grafting for esophageal cancer at institutions accredited or partially accredited by the Japan Esophageal Society were reported in 2020 [88]. The total number of patients was 41, and of these, 21 patients underwent preoperative prophylactic stent grafting. Radical resection was achieved, and some patients survived for a long time after surgery. Further accumulation of cases is expected in the future.

On the other hand, attention should be paid to the risk of development of complications of stent grafting. According to the 2020 Guideline on Diagnosis and Treatment of Aortic Aneurysm and Aortic Dissection, complications after thoracic aortic stent grafting include: (1) acute aortic syndrome represented by retrograde type A aortic dissection, (2) endoleak, (3) stroke, (4) spinal cord disorder, (5) access trouble, and (6) localized disseminated intravascular coagulopathy (consumptive coagulopathy). Particularly for (1), (3), and (4), which directly affect the life prognosis and QOL, it is considered necessary to identify high-risk patients preoperatively and adopt preventive measures intraoperatively and postoperatively. Furthermore, emergency stent grafting can be performed only in limited institutions and may be expensive; therefore, currently, this treatment should be selected very carefully according to individual case needs, taking into account patient preferences.

## Diagnosis and treatment of Barrett’s esophagus and esophageal adenocarcinoma

### Summary

Barrett’s esophagus is characterized by the replacement of the normal squamous epithelium of the distal esophagus with columnar epithelium. Barrett’s mucosa is endoscopically recognizable columnar epithelium extending from the stomach to the esophagus and does not require histological confirmation of specialized columnar epithelial metaplasia. Identification of the esophagogastric junction is required for the diagnosis of Barrett’s esophagus, and the endoscopically identifiable distal end of the lower esophageal palisade vessels is defined, in principle, as the esophagogastric junction. The definition of Barrett’s esophagus varies somewhat between Japan and Europe/the United States, in that the proximal end of the longitudinal gastric folds is defined as the esophagogastric junction in Europe/the United States and the presence of the specialized columnar epithelium is essential in Europe/the United States, excluding the United Kingdom [4–11] [92–99]. In Japan, Barrett’s esophagus with a circumferential length of ≥ 3 cm is defined as long-segment Barrett’s esophagus (LSBE), while in Europe and the United States, Barrett’s esophagus is defined as LSBE when the maximum segment length is ≥ 3 cm. Furthermore, length of Barrett’s esophagus is not defined in Japan, while columnar epithelium < 1 cm is not included in Barrett’s esophagus in many guidelines in Europe and the United States. Since all the references cited in this section used Europe/the United States definition, these guidelines refer to LSBE and short-segment Barrett’s esophagus (SSBE) based on the Europe/the United States definition of maximum length of 3 cm.

Barrett’s esophagus is characterized by at least one of the following histological findings: (1) esophageal gland ducts in the mucosa beneath the columnar epithelium or esophageal glands proper in the submucosa; (2) squamous islands within the columnar epithelium; and (3) double muscularis mucosae beneath the columnar epithelium. Barrett’s esophagus is a precursor condition of esophageal adenocarcinoma (EAC). In Europe and the United States, the histopathologic diagnosis is made using the modified Vienna classification, which defines low-grade dysplasia (LGD) and high-grade dysplasia (HGD), which are not used in Japan. LGD corresponds to adenoma or well-differentiated adenocarcinoma with low-grade atypia (noninvasive) in Japan and HGD corresponds to adenocarcinoma with high-grade atypia (noninvasive). Early, superficial, and advanced cancers are defined in the same manner as for the case of esophageal squamous cell carcinoma, in general, but the deep muscularis mucosae is handled as the original muscularis mucosae.

EAC is treated in accordance with the treatment strategies for esophageal squamous cell carcinoma. Endoscopic resection is performed for lesions preoperatively diagnosed as cT1a cancer, and when the depth of tumor invasion is found to be pT1a-SMM (confined to the columnar epithelial layer or superficial muscularis mucosae) or pT1a-LPM (beyond the superficial muscularis mucosae, but not reaching the deep muscularis mucosae) by pathological assessment after resection, the lesion is expected to be cured by endoscopic resection. Even if the depth of invasion is found to be pT1a-DMM (invading the deep muscularis mucosae) after resection, the risk of recurrence is low, unless there is vascular invasion or a poorly differentiated component.
